# Robust large-scale clustering based on correntropy

**DOI:** 10.1371/journal.pone.0277012

**Published:** 2022-11-04

**Authors:** Guodong Jin, Jing Gao, Lining Tan

**Affiliations:** Rocket Force Engineering University, Xian, Shannxi, China; Sant Longowal Institute of Engineering and Technology, INDIA

## Abstract

With the explosive growth of data, how to efficiently cluster large-scale unlabeled data has become an important issue that needs to be solved urgently. Especially in the face of large-scale real-world data, which contains a large number of complex distributions of noises and outliers, the research on robust large-scale real-world data clustering algorithms has become one of the hottest topics. In response to this issue, a robust large-scale clustering algorithm based on correntropy (RLSCC) is proposed in this paper, specifically, k-means is firstly applied to generated pseudo-labels which reduce input data scale of subsequent spectral clustering, then anchor graphs instead of full sample graphs are introduced into spectral clustering to obtain final clustering results based on pseudo-labels which further improve the efficiency. Therefore, RLSCC inherits the advantages of the effectiveness of k-means and spectral clustering while greatly reducing the computational complexity. Furthermore, correntropy is developed to suppress the influence of noises and outlier the real-world data on the robustness of clustering. Finally, extensive experiments were carried out on real-world datasets and noise datasets and the results show that compared with other state-of-the-art algorithms, RLSCC can improve efficiency and robustness greatly while maintaining comparable or even higher clustering effectiveness.

## Introduction

As the core of artificial intelligence and data science, machine learning is a discipline that aims at developing learning algorithms that build models from data (experience). In the past decades, machine learning has made great progress, and abundant techniques based on it have emerged. These techniques have played an important role in various practical applications, such as image processing [1–5], environmental monitoring [6–14], and data mining [15–25]. Among these techniques, clustering is currently one of the most popular topics in machine learning, which can automatically divide unlabeled data into different groups (clusters). In the past decades, scholars have proposed lots of impressive works. However, with the advent of the information age, clustering is bothered by several challenges. On the one hand, with the exponential rise of data, conventional clustering algorithms are finding it challenging to deal with these massive amounts of unlabeled data. The issue of how to efficiently cluster these massive amounts of unlabeled data has emerged as a critical challenge in unsupervised learning. On the other hand, in real-world clustering activities, most data contain various complex noises and outliers, which have a substantial negative impact on clustering robustness. Hence another significant problem that we should be concerned with is how to enhance the robustness of clustering algorithms in the face of real-world data. Based on the above-mentioned challenges and problems, researchers have made a lot of efforts to find a way out.

To improve the efficiency of clustering large-scale data, many accelerated clustering algorithms have been proposed with different strategies. They can be divided into k-means-based methods [15–19] and anchor graph-based methods [20–25]. As the most common acceleration algorithm, the k-means-based algorithm, which is proved to be equivalent to the algorithm based on matrix factorization, has linear computational complexity and better clustering performance. For example, FNMTF [16] and LP-FNMTF [16] proposed by Wang et al. directly constrain the factor matrix as the cluster indicator matrix to avoid additional operations when the optimization is completed. Furthermore, on this basis, Han et al. proposed a more efficient algorithm named BKM [17] to further constrain the absorption factor to a diagonal matrix to reduce the computational complexity. These k-means-based algorithms meet the efficiency requirements for processing large-scale data to a certain extent, but their direct processing of the original data makes their efficiency very sensitive to the data dimension. When the data dimension is high, their efficiency will decrease significantly [26]. As for the anchor graph-based methods, they are inspired by the idea of spectral clustering and construct anchor graphs instead of traditional full sample graphs to reduce computational complexity. Compared with traditional spectral clustering, anchor graph-based methods can greatly improve clustering efficiency while maintaining comparable clustering effectiveness, but they are still time-consuming due to the large amount of time needed to process the obtained anchor graphs. For example, ULGE [20] uses an effective method to construct a similarity matrix and then efficiently performs spectral analysis. FSCAG [21] constructs an anchor graph that takes into account spectral and spatial characteristics and performs spectral analysis to process large-scale hyperspectral images. SCHBG [22] explores the pyramid structure by a novel type of spectral clustering based on hierarchical bipartite graphs is proposed. Most of the above-mentioned anchor graph-based algorithms improve efficiency by optimizing the constructing anchor graph part, but they still have high complexity when performing spectral analysis on the obtained graphs, so it is difficult to directly apply them to those large-scale clustering tasks with higher efficiency requirements. Based on this, it is still urgently needed to develop an efficient large-scale clustering algorithm that is insensitive to data dimensions.

As for improving the robustness of real-world data clustering tasks, it is currently widely adopted to use the robustness norm to measure the error between the original data and the reconstructed representation. For example, the *L*_1_-norm-based methods [27, 28] and the method based on the *L*_21_-norm-based methods [29–31]. LSSC [27] uses the *L*_1_-norm to define a sparse coding problem to improve the robustness of the representation, RDCF [28] uses the *L*_1_-norm to minimize the error before and after the conceptual decomposition, and the *L*_21_-norm is used to select features to constrain row sparsity by enhancing the matrix and constrain the errors of the subspace representation and the original data in LSS [29] and LRR [30], respectively. Although these algorithms based on *L*_1_-norm and *L*_21_-norm can suppress simple noise better, their robustness will be significantly reduced when the noise distribution is more complex. Recently, correntropy [32], a robust local measurement criterion in information theory learning (ITL), has been introduced into clustering and has achieved good robustness [33–38], such as GCCF [34], CHNMF [35] and CSNMF [36]. However, they cannot be applied to large-scale clustering tasks due to their square or even cubic complexity. Therefore, how to introduce correntropy into large-scale real-world data clustering task to improve clustering robustness has become an important task at present.

To address the above problems, we propose a robust large-scale clustering algorithm based on correntropy (RLSCC). In the RLSCC model, for improving efficiency, pseudo-labels generated by k-means rather than the original data are utilized as the input of subsequent spectral clustering which greatly reduces the data scale involved in subsequent operations. Then anchor graph clustering instead of traditional spectral clustering is performed based on the obtained pseudo-labels to directly get the sample category which further accelerates the model. In terms of robustness, correntropy is applied in the model to suppress the impact of complex noises and outliers. The main contributions of this paper are summarized as follows:

- A novel robust large-scale clustering algorithm based on correntropy (RLSCC) is proposed in this paper. Compared with most accelerated methods which are mainly k-means-based methods and anchor graph-based methods, RLSCC is much more insensitive to data dimensions than k-means-based methods due to the implementation of pseudo-labels and graph learning while saving more time of subsequent spectral analysis than anchor graph-based methods by directly getting the sample class. Furthermore, correntropy is applied in our model to improve robustness.
- A novel optimization strategy based on half-quadratic (HQ) minimization technique [39–41] is proposed in this paper to solve the non-convex objective function of RLSCC owing to the introduction of correntropy, which can improve the efficiency as well by a few number of iterations. In addition, the complexity and parameter sensitivity of RLSCC are also analyzed.
- Extensive experiments have been performed on different real-world datasets and the results show that compared with the current mainstream fast clustering, RLSCC can efficiently obtain better performance than these algorithms.

The remainder of this paper is organized as follows:A novel robust large-scale clustering method named RLSCC is proposed in Section II. An iterative strategy is proposed for solving RLSCC and its computational complexity analysis is shown in Section III. Then, Section IV shows some experiment details and Section V is the conclusion.

## Methodology

To improve the clustering efficiency and robustness of large-scale real-world data clustering tasks, we propose a robust large-scale clustering method based on correntropy (RLSCC). This section will give a detailed description of the process of the RLSCC model.

### Pseudo-labels generation

Consider a data matrix $X\in R^{N\times D}$, where *N* is the number of samples and *D* is the number of dimensions. Put it into k-means model as follows:

$$\begin{array}{c} \min_{W\in \text{IND},C}{\left\|X-\text{WC}\right\|}_{F}^{2} \end{array}$$ (1)

where $W\in R^{N\times C}$ is a indicator matrix where **W**_*i*, *j*_ = 1 if *i*th sample is clustered into category *j*th otherwise **W**_*i*, *j*_ = 0, $C\in R^{C\times D}$ is the cluster center matrix, whose each row represent a cluster center.

After we get **W** based on **X** from k-means, **W** is regarded as pseudo-labels to participate in the follow-up process. This step successfully compress the original data with *N*×*D* scale into a small-scale data with only *N*×*C* scale, avoiding the high computational complexity required to directly perform spectral clustering on the original data. Furthermore, by applying the obtained pseudo-labels, RLSCC can inherit the advantages of k-means clustering, which can improve the effectiveness of clustering to a certain extent compared to simple spectral clustering.

### Anchor graph learning

Spectral clustering can often obtain better clustering effectiveness because it is not limited by the sample space shape and the use of sample spatial geometric information, but traditional spectral clustering is often difficult to be applied in large-scale clustering tasks due to its high computational complexity which usually is quadratic or cubic [42]. Based on this problem, anchor strategy has been developed and has been widely used in many graph learning works. This subsection gives some details about the process of anchor graph learning.

#### Anchors generation

There are currently two main methods for generating anchors which are random sampling and k-means, respectively. Because k-means can often provide better clustering performance under the same number of anchors than random sampling, this work uses k-means to coarsely cluster the original data to get representative anchors for the graph constructing part of spectral clustering.

#### Anchor graph construction

After getting all anchors defined as **s**_**1**_,…, **s**_**M**_ in our work, the anchor graph needs to be constructed between the samples set and the anchors set. Traditional anchor graph construction methods usually include:1) Calculate the distance between all points in the samples set and all points in the anchors set to directly obtain a distance matrix; 2) Set a fixed threshold, let the distance less than it be 1, and the rest are all 0; 3) Set a fixed threshold, the distance less than it remains value, and the rest is set to 0. Although these methods can obtain anchor graph and have applied in many cases, their exploration of the geometric structure of the sample space is limited. In our work, following [43, 44], a normalized KNN anchor graph is constructed by using the first k-nearest neighbors of a fixed sample as follows:

$$\begin{array}{c} A_{ij}^{\left(v\right)}=\frac{\hat{D}\left(x_{i}^{\left(v\right)},s_{k+1}^{\left(v\right)}\right)-\hat{D}\left(x_{i}^{\left(v\right)},s_{j}^{\left(v\right)}\right)}{\sum_{\hat{j}=1}^{k}\left(\hat{D}\left(x_{i}^{\left(v\right)},s_{k+1}^{\left(v\right)}\right)-\hat{D}\left(x_{i}^{\left(v\right)},s_{\hat{j}}^{\left(v\right)}\right)\right)}A_{ij}=\frac{\hat{D}\left(x_{i},s_{k+1}\right)-\hat{D}\left(x_{i},s_{j}\right)}{\sum_{\hat{j}=1}^{k}\left(\hat{D}\left(x_{i},s_{k+1}\right)-\hat{D}\left(x_{i},s_{\hat{j}}\right)\right)} \end{array}$$ (2)

where $\overset{´}{D}\left(x,s\right)$ is a sort function that can sort the distance in ascending order, and it satisfies $\overset{´}{D}\left(x,s\right)=\phi \left(D\left(x,e\right)\right)$, and $D\left(x,e\right)=||x-{e||}_{2}^{2}$. The property of $\sum_{j=1}^{m}s_{ij}=1$ can get a more meaningful clustering performance.

### Anchor graph based clustering with pseudo-labels

To inherit the high efficiency of k-means and the high effectiveness of graph-based clustering, a fast clustering model is proposed in this subsection, which uses the correntropy to minimize the clustering results of k-means and graph-based clustering to ensure the clustering effectiveness and robustness, while greatly improving the efficiency of clustering. The objective function of RLSCC can be defined as the following form:

$$\begin{array}{c} \max_{U_{p}}Q(U_{p})=\sum_{i=1}^{N}G(\sqrt{\sum_{i=1}^{K}{\left(W_{ij}-{\left(Z_{p}U_{p}\right)}_{ij}\right)}^{2}}) \end{array}$$ (3)

where **G**(⋅) represents the kernel function of correntropy, and **Z**_**p**_ is the first *p* columns of **Z** which denotes the best represent the structure of the sample graph. **Z** can be defined as:

$$\begin{array}{c} \left[Z,V,H^{⊤}]=svd(L\right) \end{array}$$ (4)

where **L** is the Laplacian matrix of anchor graph **A** and it can be defined as:

$$\begin{array}{c} L=I-D^{-1/2}SD^{-1/2} \end{array}$$ (5)

where **S** = **A**^⊤^ **A** denotes the similarity matrix of **A**, and **D** is the degree matrix of **A** satisfies *d*_*ii*_ = ∑_*j*_ *s*_*ij*_.

## Optimization and analysis

### Optimization

In this subsection, an iterative optimization method is proposed to solve the objective function. Note that Eq (3) is a non-convex functions. To bring the optimization problem into a convex situation, we use half-quadratic technology to transform Eq (3) into the following formula:

$$\begin{array}{c} \min_{U_{p},V}O=\sum_{i=1}^{N}(v_{i}(\sum_{i=1}^{K}{\left(W_{ij}-{\left(Z_{p}U_{p}\right)}_{ij}\right)}^{2})) \end{array}$$ (6)

where **V** is a diagonal matrix, and its *i*th diagonal element **V**_*ii*_ = *v*_*i*_ can be given as:

$$\begin{array}{c} \begin{array}{cc} v_{i} & =\frac{G(\sqrt{\sum_{i=1}^{K}{\left(W_{ij}-{\left(Z_{p}U_{p}\right)}_{ij}\right)}^{2}})}{\sigma^{2}}\\ & =\frac{\text{exp}(-(\sum_{i=1}^{K}{\left(W_{ij}-{\left(Z_{p}U_{p}\right)}_{ij}\right)}^{2})/2\sigma^{2})}{\sigma^{2}} \end{array} \end{array}$$ (7)

where *σ* is a free parameter that controls the robustness of the correntropy.

Now, the objective function Eq (6) can be solved directly, and the proposed optimization formulation contains two variants totally. Here, we fix one of them and update the other one. In practice, the iterative optimization performs two steps:**V**-step and **U**_**p**_-step. The specific steps are as follows:

#### V-step

Fixing **U**_**p**_, **V** can be updated as the following formula:

$$\begin{array}{c} V_{ii}=v_{i}=\text{exp}(-\frac{\sum_{i=1}^{K}{\left(W_{ij}-{\left(Z_{p}U_{p}\right)}_{ij}\right)}^{2}}{2\sigma^{2}})/\sigma^{2} \end{array}$$ (8)

#### U_p_-step

When **V** is fixed, Eq (6) can be transformed into:

$$\begin{array}{c} \begin{array}{cc} \min_{U_{p}}O & =Tr({\left(W-Z_{p}U_{p}\right)}^{⊤}V(W-Z_{p}U_{p}))\\ & =Tr(W^{⊤}VW)-2Tr(U_{p}^{⊤}Z_{p}^{⊤}VW)+Tr(U_{p}^{⊤}Z_{p}^{⊤}VZ_{p}U_{p}) \end{array} \end{array}$$ (9)

Assuming that $\Phi =\left[\Phi_{ij}\right]\in R^{n\times c}$ are Lagrange multipliers, the Lagrange function of Eq (6) can be expressed by the following formula:

$$\begin{array}{c} L=Tr(W^{T}VW)-2Tr(U_{p}^{⊤}Z_{p}^{⊤}VW)+Tr(U_{p}^{⊤}Z_{p}^{⊤}VZ_{p}U_{p})+Tr(\Phi U_{p}) \end{array}$$ (10)

Then, let L find the partial derivative of **U**_**p**_, we can get:

$$\begin{array}{c} \frac{\partial L}{\partial U_{p}}=-2(Z_{p}VW)+2(Z_{p}^{⊤}VZ_{p}U_{p})+\Phi \end{array}$$ (11)

Using the KKT condition (**Φ**_*ij*_ **U**_**p**__*ij*_ = 0), we have:

$$\begin{array}{c} -2{\left(Z_{p}^{⊤}VW\right)}_{ij}U_{p}ij+2{\left(Z_{p}^{⊤}VZ_{p}U_{p}\right)}_{ij}U_{p}ij+\Phi_{ij}U_{p}ij=0 \end{array}$$ (12)

Subsequently, we can get the following updated iteration rules of **U**_**p**_:

$$\begin{array}{c} U_{p_{ij}}\leftarrow U_{p_{ij}}\frac{{\left(Z_{p}^{⊤}VW\right)}_{ij}}{{\left(Z_{p}^{⊤}VZ_{p}U_{p}\right)}_{ij}} \end{array}$$ (13)

**Algorithm 1** Algorithm to solve the RLSCC model of Eq (3)

**Require:** The original data $X\in R^{N\times D}$, the number of anchors *M*, the Gaussian kernel bandwidth *σ*, and the number of *p* columns of **Z**.

**Ensure:** The cluster indicator matrix **Y**.

 1: Generate *M* anchors from *N* samples by using k-means.

 2: Construct anchor graph by Eq (2).

 3: Initialize **U**_**p**_ to a random non-negative matrix, and get **Z**_**p**_ from Eq (4).

 4: **while** not converge **do**

 5: Update **V** by Eq (8).

 6: Update **U**_**p**_ by Eq (13).

 7: **end while**

By iteratively updating **V** and **U**_**p**_ until the objection function converges, we can directly obtain the category to which each sample belongs from the optimal probabilistic clustering matrix **Y** = **Z**_**p**_ **U**_**p**_. The details of the process are shown in Algorithm 1.

### Computational complexity

The computational complexity of RLSCC is mainly composed of the following parts:anchors generation, anchor graph construction, and iterative optimization. The details of the complexity of these parts are as follows:

1. The complexity of *O*(*NMDT*_1_) is needed to use k-means to generate *M* anchors from *N* samples, and *T*_1_ is the iteration number of k-means.
2. *O*(*NMD*) complexity is desired when constructing anchor graph between *N* samples and *M* anchors by utilizing Eq 2.
3. *O*((*NC*^2^+ *NCp*)*T*) is needed when optimizing. To be precise, *O*(*NC*^2^) is needed to solve **V**, *O*(*NCp*) is demanded to update **U**_**p**_, and *T* is the number of iteration of the objective function to convergence.

Generally speaking, the overall computational complexity of RLSCC is *O*(*NMDT*_1_+ (*NC*^2^+ *NCp*)*T*). Since *M*, *D*, *p*, *C*, *T*_1_, and *T* are much smaller than *N* when dealing with large-scale data, the complexity of RLSCC can be approximately *O*(*N*). In particular, when the dimension of the data is large, RLSCC can still maintain a low computational complexity because it is independent of the dimension in the optimizing iteration part when solving the objective function.

## Experiments

In this section, we give a comparison of the performance among RLSCC and six states-of-the-art algorithms (CF [45], LPFNMTF [16], LRS [46], LSSC [27], GCCF [33], EC [47]) on six datasets (TDT2 [48], Mnist [49], Corel [50, 51], Motper1 and Motper2 (http://www.escience.cn/people/fpnie/index.html), Corel [50, 51], and USPS [49]). Table 1 shows some properties of the six datasets. And also to validate the robustness of our methods, numerous experiments in noisy datasets are carried out. Specifically, six different metrics:ACC [52], NMI [53], Purity [54], ARI [55], F-score [56] and Precision [57] are used to verify the effectiveness and robustness of RLSCC.

**Table 1:** Datasets description.

Datasets	Corel	Mnist	Motper1	Motper2	USPS	TDT2
*Samples*	5000	6996	5000	5000	9298	9394
*Dimesion*	423	784	48	48	256	36771
*Classes*	50	10	5	5	10	30
*Type*	Image	Image	Image	Image	Image	Text

### Compared methods and parameter setting

Six states-of-the-art clustering algorithms (CF, LPFNMTF, LRS, LSSC, GCCF, EC) are presented as compared methods in this part to verify the advantages of our algorithm over the mainstream clustering algorithms for large-scale data. A brief introduction of the comparison algorithms are outlined as follows:

**CF**’s full name is concept factorization. It models each concept as a linear combination of the data points, and each data point as a linear combination of the concepts. Differing from the method of clustering based on non-negative matrix factorization (NMF) [58], this method can be applied to data with negative values and can be implemented in the kernel space.

**LPFNMTF** is a local preserving regularization method based on fast non-negative matrix factorization. By using manifold regularization, this method can realize the geometric constraints on the two factorization factor matrices. What’s more, an optimization algorithm for LPFNMTF is proposed, which greatly improve efficiency by reducing the multiplication of matrix.

**LRS** is a new subspace clustering model to cluster data which is drawn from multiple linear or affine subspaces. Instead of using two steps’ algorithm (building the affinity matrix and spectral clustering). It directly learns the different subspaces’ indicator so that low-rank based different groups are obtained clearly. What’s more, this method use Schatten *p*-norm [59] to relax the rank constraint instead of using trace norm for better approximation of the low-rank constraint.

**LSSC** is a large-scale sparse clustering algorithm, using *L*_1_-norm for regularization to exploit matrix sparsity and obtain more robustness. Meanwhile, the model uses nonlinear approximation and dimension reduction techniques to further speed up the sparse coding algorithm, which brings high efficiency.

**GCCF** is a clustering algorithm based on correntropy, which introduces the correntropy technique into the clustering analysis for the first time, and uses the correntropy to good suppression of nonlinear and non-Gaussian noise to improve the accuracy of clustering results.

**EC**’s full name is extreme clustering and it is a clustering method via density extreme points proposed for overcoming the drawbacks of peak clustering [60]. The theme of extreme clustering is to identify density extreme points to find cluster centres. What’s more, to guarantee the robustness, a noise detection module is also introduced to eliminate the influence of noisy data points.

In Table 2, we summarize the computational complexity of the compared methods. Some common notions for all methods, including the number of samples, classes, dimensions, and optimization iterations, are represented as *N*, *C*, *D*, *T*, respectively. Meanwhile, there are some method-specific notations whose meanings are as follows: *M*_1_ in LPFNMTF indicates the additional dimension number introduced by NMTF, *M*_2_, *p*, and *T*_1_ in LSSC indicate the selected clustering centers for nonlinear approximation, the number of leading eigenvectors, and the iteration number of k-means, respectively.

**Table 2:** Computational complexity summary.

Method	Computational Complexity
*CF*	*O*(*N* ^2^ *CT*+ *N* ^2^ *D*)
*LPFNMTF*	$O(\left(NM_{1}D+NM_{1}C+NC^{2}+M_{1}C^{2}+M_{1}^{2}D+C^{3}+M_{1}^{3}\right)T)$
*LRS*	*O*(*NCD* ^2^ *T*)
*LSSC*	*O*((*NCD*+ *NM*_2_ *D*)*T*_1_+ *NC* ^2^+ (*p*^3^+ *Np* ^2^+ *NCp*+ *Cp* ^2^)*T*)
*GCCF*	*O*((*N* ^2^ *C*+ *N* ^2^ *D*+ *NCD*)*T*)
*EC* ^@^	*O*(*N* ^2^ *D*)
*Ours*	*O*(*NMDT*_1_+ (*NC* ^2^+ *NCp*)*T*)

^@^ Here we only list the main complexity of EC since the uncertain complexity (but less than or equal to *O*(*N*^2^)) in extreme points identification.

For these compared methods which owns parameters (LPFNMTF, LSSC, and GCCF) affecting the clustering performance, our settings are as follows: LPFNMTF and GCCF own two parameters including the regularization parameter *λ* and the number of nearest neighbors. For the two methods, we select *λ* from the set {1*e*1, 1*e*2, 1*e*3} and *p* from the set {3, 5, 7} to tune the results to the optimal results; For LSSC, the regularization parameter is set as 0.1 as author’s advice. All the compared methods are tune to their best based on our capability.

### Clustering results

In this part, we adopt six widely used metrics, which contain ACC, NMI, Purity, ARI, F-score, and Precision, to verify the performance of RLSCC and the compared methods on six datasets. For all clustering methods, the larger values of the metrics are expected to achieve better performance. To be fair, all experiments were performed five times on a laptop computer configured as a 16.0GB 3.20GHz AMD Ryzen CPU 5800H, at Matlab 2020b (64bit), and the mean values were recorded and the optimal and suboptimal results are marked in bold. Meanwhile, the mark and star indicate the computing time greater than 3 hours and the memory overflow when performing the experiment, respectively.

For the clustering efficiency, it can be observed from Table 2 in theory that compared to other methods, the complexity of the proposed method is less sensitive to the number of samples and the number of dimensions. And from the aspect of practice, Table 3 shows the computational time of various methods on different datasets, we can observe that RLSCC can achieve the same or better level of efficiency as high-efficient clustering methods like LPFNMTF, LSSC, and EC and hundreds of times faster than CF and LRS. What’s more, on TDT2, which is a high-dimensional data, the computational time of RLSCC is much more stable compared with these k-means based methods (LPFNMTF, GCCF, and CF), showing RLSCC is much more insensitive to data dimensions due to the implementation of pseudo-labels and graph learning. And compared with the robust methods (LRS, GCCF, and LSSC), especially when compared with GCCF which also uses correntropy to suppress noise, RLSCC shows more efficiency. The high efficiency of RLSCC benefits mainly from the pseudo-labels generation and anchors generation step which inherit the advantages of k-means and anchor-based anchor-based spectral clustering respectively. Concretely, the implementation of pseudo-labels and graph learning makes RLSCC insensitive to data dimensions while the strategies of anchor and directly obtaining the getting the sample classes further improve the efficiency.

**Table 3:** Running time comparison on datasets (seconds).

Method	Corel	Mnist	Motper1	Motper2	USPS	TDT2
*CF*	182.5865	434.0780	157.3665	153.3496	1078.9258	-
*LPFNMTF*	42.8330	39.2015	2.9533	2.6766	23.8256	*
*LRS*	1731.1413	1259.4178	319.3305	319.7345	703.6245	*
*LSSC*	3.3861	**7.1655**	2.9161	2.8530	**9.8471**	**47.6587**
*GCCF*	49.4777	136.3959	18.5541	17.1630	66.1137	*
*EC*	**3.2107**	9.4889	**2.3432**	**2.3413**	10.3423	913.2778
*Ours*	**3.0963**	**5.4838**	**2.4273**	**2.3122**	**7.8403**	**57.1852**

As for the clustering effectiveness, Tables 4–6 show the effectiveness of RLSCC and the compared methods on six datasets. As presented in the tables, RLSCC can achieve the top two effectiveness in the six metrics and on six datasets. Especially, in some cases such as, on Corel, Mnist, and USPS datasets, ACC, NMI, Purity, ARI, and F-score of RLSCC are averagely higher than the suboptimal results:20.3%, 16.0%, 9.2%, 38.9%, 19.2%, and 33.4% respectively, which demonstrates the high effectiveness of RLSCC. When combining all the tables, it can be observed that RLSCC can improve the clustering efficiency greatly while guaranteeing comparable or even better clustering effectiveness.

**Table 4:** Clustering results comparison on Corel and Mnist.

Method	Corel						Mnist					
	ACC	NMI	Purity	ARI	F-score	Precision	ACC	NMI	Purity	ARI	F-score	Precision
*CF*	**0.1080**	**0.1636**	**0.1206**	**0.0269**	**0.0597**	**0.0351**	**0.3320**	**0.2036**	**0.3158**	**0.1217**	**0.2472**	**0.1734**
*LPFNMTF*	0.0542	0.0694	0.0694	-0.0001	0.0197	0.0197	0.1157	0.0026	0.0026	0.0000	0.1000	0.1002
*LRS*	0.0812	0.1041	0.0878	0.0137	0.0446	0.0282	0.1431	0.0195	0.1431	0.0065	0.1804	0.1032
*LSSC*	0.0200	0.0000	0.0200	0.0000	0.0388	0.0198	0.1125	0.0000	0.1125	0.0000	0.1821	0.1002
*GCCF*	0.0200	0.0000	0.0200	0.0200	0.0388	0.0198	0.1136	0.0010	0.1136	0.1136	0.1820	0.1002
*EC*	0.0368	0.0292	0.0426	0.0003	0.0390	0.0200	0.1394	0.0612	0.1734	0.0042	0.1820	0.1021
*Ours*	**0.1144**	**0.1733**	**0.1210**	**0.0340**	**0.0635**	**0.0412**	**0.3699**	**0.2974**	**0.4238**	**0.2051**	**0.2963**	**0.2586**

**Table 5:** Clustering results comparison on Motper1 and Motper2.

Method	Motper1						Motper2					
	ACC	NMI	Purity	ARI	F-score	Precision	ACC	NMI	Purity	ARI	F-score	Precision
*CF*	0.4052	0.0852	0.2699	0.0662	0.3679	0.2309	0.2834	0.0802	0.3039	0.0442	0.3360	0.2209
*LPFNMTF*	0.2104	0.0011	0.0011	0.0001	0.1999	0.1999	0.2122	0.0007	0.0007	-0.0003	0.1996	0.1996
*LRS*	0.2630	0.0159	0.2630	0.0119	0.2883	0.2059	0.2024	0.0010	0.2024	0.0000	0.3326	0.1998
*LSSC*	0.2506	0.0140	0.2506	0.0114	0.3086	0.2051	0.3698	0.1301	0.3792	0.1288	0.3742	0.2655
*GCCF*	0.2000	0.0000	0.2000	0.2000	0.3331	0.1998	0.2000	0.0000	0.2000	0.2000	0.3331	0.1998
*EC*	**0.4930**	**0.5062**	**0.7330**	**0.3521**	**0.4880**	**0.4648**	**0.5174**	**0.5852**	**0.9700**	**0.5182**	**0.5780**	**0.9309**
*Ours*	**0.5266**	**0.3950**	**0.5824**	**0.3054**	**0.4493**	**0.4336**	**0.5656**	**0.4277**	**0.5656**	**0.3251**	**0.4734**	**0.4305**

**Table 6:** Clustering results comparison on USPS and TDT2.

Method	USPS						TDT2					
	ACC	NMI	Purity	ARI	F-score	Precision	ACC	NMI	Purity	ARI	F-score	Precision
*CF*	**0.3385**	0.2464	0.3745	0.1617	0.2880	0.2115	-	-	-	-	-	-
*LPFNMTF*	0.1120	0.0020	0.0020	0.0000	0.1036	0.1075	*	*	*	*	*	*
*LRS*	0.2206	0.0878	0.2629	0.0561	0.1920	0.1434	*	*	*	*	*	*
*LSSC*	0.1670	0.0000	0.1670	0.0000	0.1941	0.1075	0.1963	0.0000	0.1963	0.0000	0.1974	0.1095
*GCCF*	0.1671	0.0001	0.1672	0.1671	0.1941	0.1075	*	*	*	*	*	*
*EC*	0.3319	**0.4010**	**0.6049**	**0.1868**	**0.3153**	**0.2145**	**0.2385**	**0.0643**	**0.2471**	**0.0131**	**0.2052**	**0.1155**
*Ours*	**0.6158**	**0.4503**	**0.6158**	**0.4184**	**0.4860**	**0.4496**	**0.5037**	**0.4093**	**0.5824**	**0.4508**	**0.5171**	**0.4689**

### Robustness analysis

**Fig 1:**
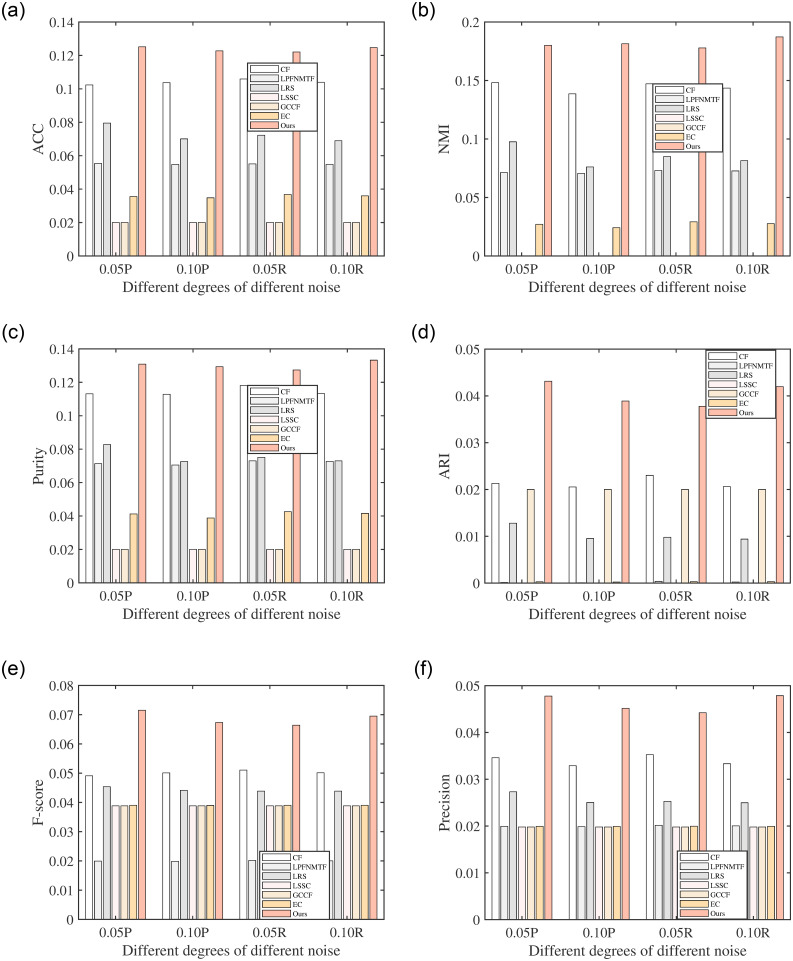
Clustering results of all algorithms with different noise on Corel. (a) ACC on Corel with different noise, (b) NMI on Corel with different noise, (c) Purity on Corel with different noise, (d) ARI on Corel with different noise, (e) F-score on Corel with different noise, and (f) Precision on Corel with different noise.

**Fig 2:**
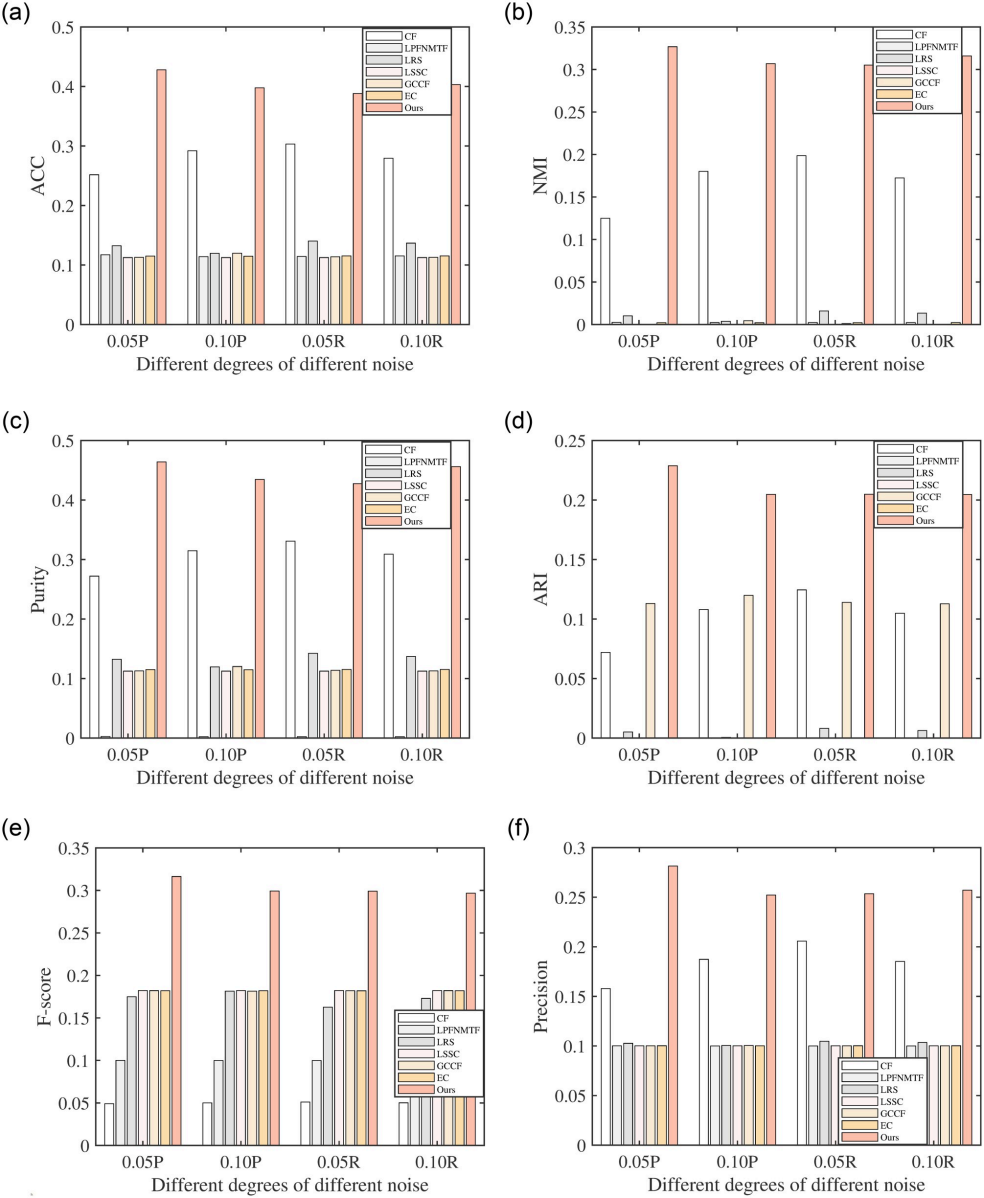
Clustering results of all algorithms with different noise on Mnist. (a) ACC on Mnist with different noise, (b) NMI on Mnist with different noise, (c) Purity on Mnist with different noise, (d) ARI on Mnist with different noise, (e) F-score on Mnist with different noise, and (f) Precision on Mnist with different noise.

As mentioned before, the introduction of correntropy can bring RLSCC resistance to various noises in real-world datasets. To verify the robustness of RLSCC, extensive experiments have been performed in eight noisy datasets. Specifically, we added different degrees (5%, 10%) of random noise and possion noise to Corel and Mnist to form different noise datasets and performed RLSCC, and compared methods on these datasets under the same experimental conditions. The results are shown in Figs 1 and 2, from which we can obtain that the performance of RSCL can be maintained at the original level. Especially, compared with LSSC which uses the *L*_1_-norm to achieve robustness, RLSCC gives better clustering performance and robustness in all cases when facing more complex (non-linear and non-Gaussian) noise, which shows the advantage of correntropy.

### Parameter analysis

**Fig 3:**
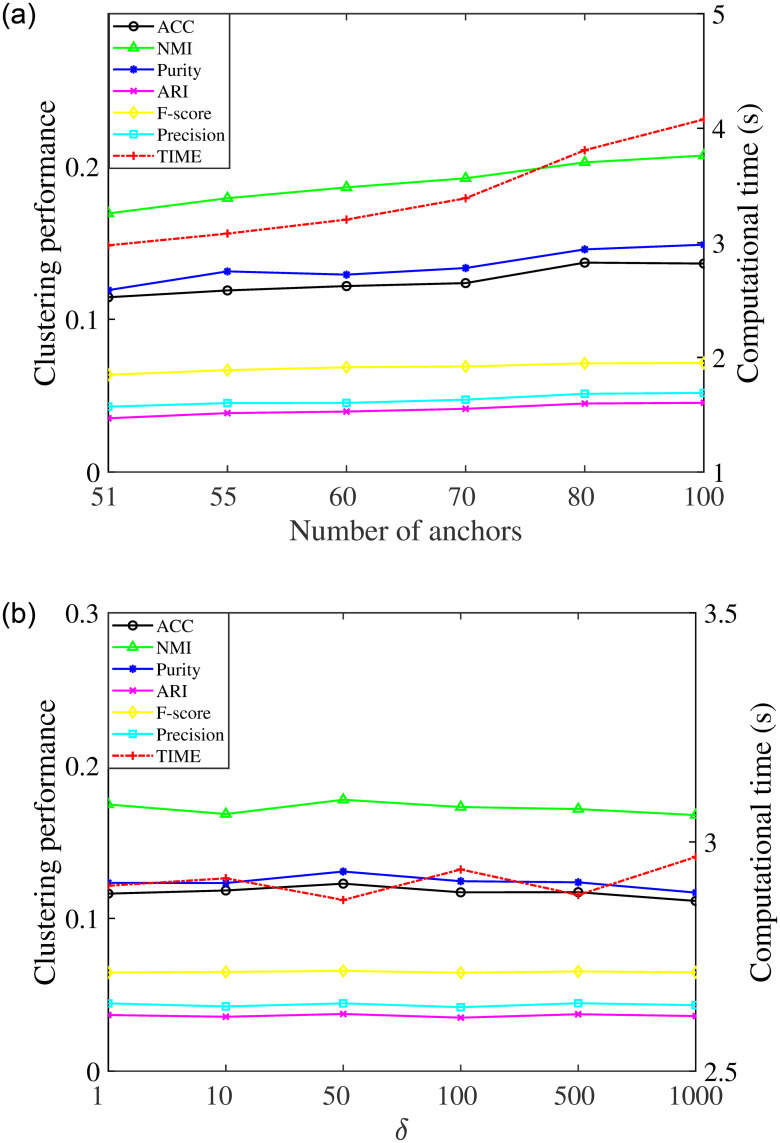
Clustering results of RLSCC with different number of anchors on Corel and Mnist. (a) Clustering results on Corel and (b) Clustering results on Mnist.

**Fig 4:**
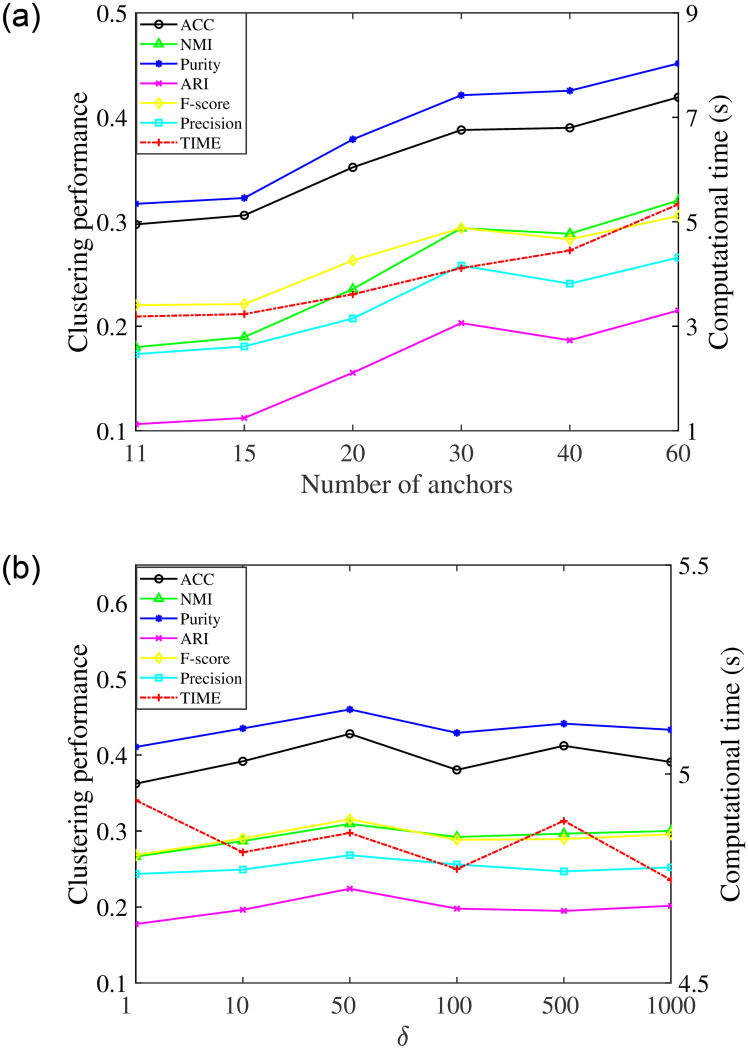
Clustering results of RLSCC with different *δ* on Corel and Mnist. (a) Clustering results on Corel and (b) Clustering results on Mnist.

There are two main parameters contained in RLSCC:the number of anchors *M*, which affect the clustering effectiveness and efficiency, and the bandwidth of the Gaussian kernel *δ*, which determines the robustness of the model. To validate the impact on the efficiency and effectiveness of these two parameters, we perform RLSCC under different parameter conditions and discuss the results in this part. The number of anchors has a huge effect on the clustering performance and efficiency. It is important to choose a suitable number of anchors to make a good trade-off between effectiveness and efficiency when performing RLSCC. To explore a proper M, extensive experiments were done using different M from the set of {*c*+1, *c*+5, *c*+10, *c*+20, *c*+30, *c*+50}, where *c* is the number of categories of the dataset. Fig 3 shows the clustering performance and computational time of different numbers of anchors when *δ* = 10. On the one hand, the clustering performance shows an overall upward trend, and the upward trend is gradually becoming slower as the number of anchors increases. On the other hand, the computational time continues to increase with the growth of the number of anchors but it in general remains at a low level. Therefore, RLSCC can give a satisfying trade-off between efficiency and effectiveness via a suitable selection of the number of anchors. As another important parameter, the bandwidth of the Gaussian kernel *δ* impacts the robustness of RLSCC. Fig 4 presents the influence of different bandwidths of the Gaussian kernel when *M* = 50 on the final clustering results and computational time from an experimental point of view. In these experiments, *δ* is selected in {1, 10, 50, 100, 500, 1000}. We can obtain that the clustering results and computational time basically hover in a certain and acceptable range with the increase of *δ*.

## Conclusion

This paper proposes a robust large-scale clustering algorithm based on correntropy (RLSCC), which inherits the low computational complexity of k-means and the high effectiveness of spectral clustering. Meanwhile, the generation of pseudo-labels and the use of anchor graphs can effectively improve the efficiency of clustering. To solve RLSSC, a new fast optimization algorithm based on half-quadratic technology is proposed, which can complete the confirmation of the sample category in a short time. Finally, extensive experiments on real-world datasets and noisy datasets show that compared to other state-of-the-art algorithms, especially when facing large-scale high-dimensional data, RLSCC can ensure higher efficiency and robustness while remaining comparable or even better clustering effectiveness. However, there are still some limitations to the present method. On the one hand, the performance of k-means is easily affected by initialization, which may affect the generation quality of pseudo-labels and anchor graphs and further affect the clustering effectiveness. On the other hand, the proposed method can not be applied to multi-view datasets, which are now common in real applications. Therefore, the future scope of the present work is to apply novel methods for pseudo-labels and anchor graph generation and to extend the work to a multi-view version.
